# Efficacy of text-message reminders on paediatric malaria treatment adherence and their post-treatment return to health facilities in Kenya: a randomized controlled trial

**DOI:** 10.1186/s12936-017-1702-6

**Published:** 2017-01-25

**Authors:** Ambrose O. Talisuna, Amos Oburu, Sophie Githinji, Josephine Malinga, Beatrice Amboko, Philip Bejon, Caroline Jones, Robert W. Snow, Dejan Zurovac

**Affiliations:** 1 KEMRI-Welcome Trust Research Programme, Nairobi, Kenya; 2 Centre for Tropical Medicine and Global Health, University of Oxford, Oxford, UK

**Keywords:** SMS, Artemether–lumefantrine, Adherence, Follow-up

## Abstract

**Background:**

Short Message Service (SMS) reminders have been suggested as a potential intervention for improving adherence to medications and health facility attendance.

**Methods:**

An open-label, randomized, controlled trial to test the efficacy of automated SMS reminders in improving adherence to artemether–lumefantrine (AL) and post-treatment attendance in comparison with standard care was conducted at four health facilities in western Kenya. Children below five years of age with uncomplicated malaria were randomized to intervention (SMS reminders) or control groups. Within each study group they were further randomized to three categories, which determined the timing of home visits to measure adherence to complete AL course and to individual AL doses. A sub-set of caregivers was advised to return to the facility on day 3 and all were advised to return after 28 days. The primary outcomes were adherence to medication and return on day 3. The primary analysis was by intention-to-treat.

**Results:**

Between 9 June, 2014 and 26 February, 2016, 1677 children were enrolled. Of 562 children visited at home on day 3, all AL doses were completed for 97.6% (282/289) of children in the control and 97.8% (267/273) in the intervention group (OR = 1.10; 95% CI = 0.37–3.33; p = 0.860). When correct timing in taking each dose was considered a criteria for adherence, 72.3% (209/289) were adherent in the control and 69.2% (189/273) in the intervention group (OR = 0.82; 95% CI = 0.56–1.19; p = 0.302). Sending SMS reminders significantly increased odds of children returning to the facility on day 3 (81.4 vs 74.0%; OR = 1.55; 95% CI = 1.15–2.08; p = 0.004) and on day 28 (63.4 vs 52.5%; OR = 1.58; 95% CI = 1.30–1.92; p < 0.001).

**Conclusions:**

In this efficacy trial, SMS reminders increased post-treatment return to the health facility, but had no effect on AL adherence which was high in both control and intervention groups. Further effectiveness studies under the real world conditions are needed to determine the optimum role of SMS reminders.

*Trial registration* ISRCTN39512726

**Electronic supplementary material:**

The online version of this article (doi:10.1186/s12936-017-1702-6) contains supplementary material, which is available to authorized users.

## Background

The expansion of network coverage and mobile phone penetration in Africa [1] has offered opportunities to improve health communication and support medical and public health practice [2]. Text messaging or Short Message Service (SMS), the widely used mobile phone function, has recently been deployed in numerous health projects across Africa [3]. Trials across the continent have shown that SMS reminders sent to patients’ mobile phones can improve adherence to antiretroviral therapy [4, 5], immunization coverage [6], blood pressure control [7], emotional outcomes after abortion [8], as well as antenatal [9], delivery [10], postpartum [11], postoperative [12], and repeat HIV test [13] attendance. Conversely, in other trials, SMS reminders were not effective in improving adherence to antiretroviral therapy or voluntary male circumcision [14, 15].

Most SMS interventions in Africa have been assessed in the management of chronic diseases and long-term therapy, while their effects on the management of acute diseases, such as malaria, have been less commonly investigated [16]. SMS reminders sent to either health workers or malaria patients and their caregivers have been suggested as a potential intervention [16–18] to improve sub-optimal caregivers’ adherence to artemisinin-based combination therapy (ACT) [19, 20] and poor outpatient attendance rates for follow-up [21]. No previous study has examined the efficacy of SMS reminders to enhance patients’ return to the health facility following malaria treatment. Two trials, showing discrepant results, tested effects of SMS reminders on patients’ adherence to ACT [22, 23].

Non-adherence to anti-malarial medicines and lack of patients’ follow-up compromises malaria case management and favours the emergence of anti-malarial resistance [24, 25]. The latter is becoming increasingly important with the risk of artemisinin resistance spreading from Southeast Asia to sub-Saharan Africa [26–28]. Early warnings of the emergence of resistance may be detected by post-treatment monitoring of the outcomes of ACT treatment [29, 30]. However, such post-treatment monitoring is only possible if patients return to the health facility when requested.

A randomized controlled trial was therefore undertaken in Kenya to assess whether SMS reminders sent to caregivers of children treated with nationally recommended ACT, artemether–lumefantrine (AL), would enhance adherence to AL therapy and would increase the rates of post-treatment return to the health facility following completion of treatment.

## Methods

### Study area

The trial was conducted at four public health facilities in Siaya County in western Kenya. Two trial sites are located in Bondo Sub-county (Bondo and Got Agulu Hospitals) and two in Rarieda Sub-county (Ndori Health Centre and Madiany Hospital). Malaria transmission in the study area is high with seasonal peaks in May–July and October–November [31]. AL has been routinely used since 2006 as the first-line treatment for uncomplicated malaria. Reports of children completing AL course ranged from 58–81% [32, 33]. A feasibility study undertaken prior to the trial found that the mobile network coverage in the study area was nearly universal with over 90% of caregivers of children with malaria having access to mobile phones and expressing willingness to receive SMS reminders about drug administration and when to return to the health facility [34].

### Study design

**Fig. 1:**
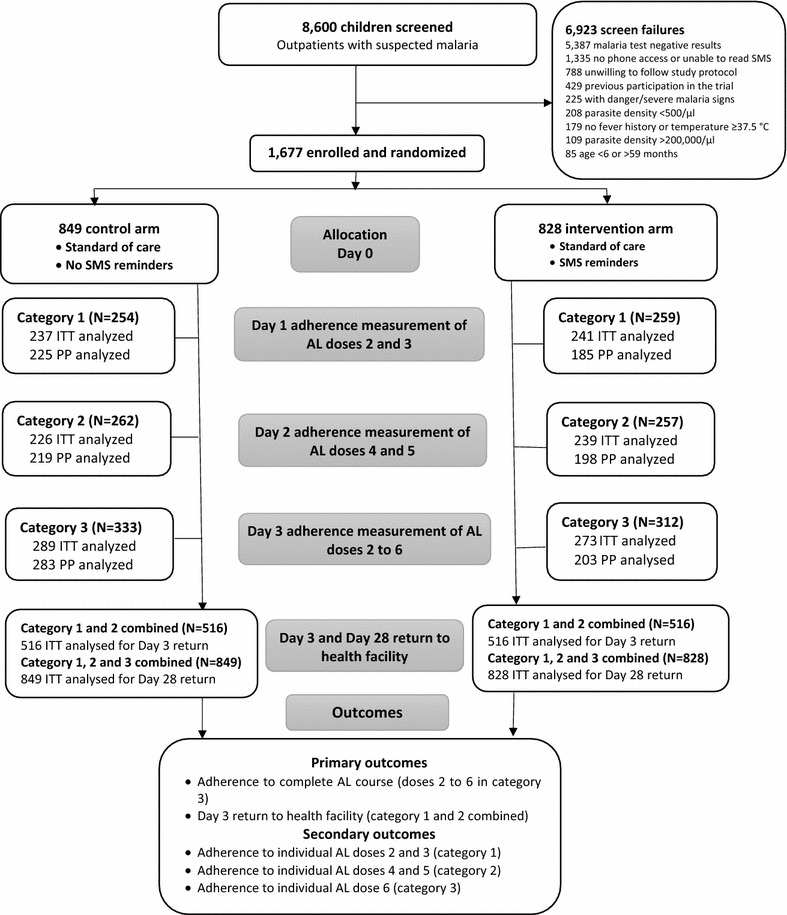
Trial profile

The study was an open-label, randomized, controlled trial testing the additional effects of SMS reminders on patients’ adherence to AL and their return to the health facility compared to the control group receiving standard care only. This was an efficacy trial where all children with uncomplicated malaria received care by study personnel in line with national guidelines [35], and with additional advice to return to the facility on day 3 and day 28 post-treatment, to facilitate resistance surveillance. Participants were randomized either to the intervention (SMS reminders) or to control groups. As a second randomization within each study group, they were further randomly assigned to three different categories, which determined the timing of home visits to measure adherence (Fig. 1). The categories to determine timing of these visits were: category 1: caregivers were visited at home on day 1 to measure adherence of the second and the third AL dose; category 2: caregivers were visited on day 2 to measure adherence of AL doses 4 and 5; and, category 3: caregivers were visited at home on day 3 after they had completed the full treatment to measure adherence to the complete course of AL (doses 2–6) and adherence to the individual dose 6. These home visits were used to mitigate the anticipated recall bias for measurements of timely adherence to individual AL doses [36]. Furthermore, caregivers in categories 1 and 2 were advised to return to the facility on day 3 and all caregivers were advised to return to the facility on day 28. Outcomes were compared across randomization groups (i.e., intervention vs control) and were assessed within applicable categories as follows: the primary outcomes were: (a) the proportion of patients adhering to complete AL course (measured among category 3); and, (b) the proportion of patients’ returning to the facility on day 3 (measured among categories 1 and 2 combined). The secondary outcomes were (a) adherences to five individual AL doses (measured among all 3 categories); and, (b) patients’ return to the facility on day 28 (measured among all 3 categories).

### Enrolment

All children suspected of malaria were screened by study clinicians at outpatient departments in the study sites and enrolled into the trial if they met all of the following inclusion criteria: age six to 59 months; weight 5 kg and above; history of fever in the previous 24 h or presence of axillary temperature ≥37.5 °C; microscopically confirmed infection of *Plasmodium falciparum* with parasitaemia between 500 and 200,000/μL; caregiver access to personal or shared mobile phone within household; caregiver ability to open and read SMS either themselves or through another person in the household; and, caregiver provision of written informed consent. The following exclusion criteria applied: presence of clinical danger signs, severe anaemia [haemoglobin (Hb) <5 g/dl] or any other severe malaria criteria; severe malnutrition (weight for height <70%); ongoing prophylaxis with drugs having anti-malarial activity such as cotrimoxazole; reported hypersensitivity to AL; presence of any concurrent illness; and, previous participation in the trial. Thick and thin blood smears were performed by the study microscopists, who counted asexual parasites per 200 white blood cells (WBCs) and calculated parasite density on the estimate of 8000 WBCs/μL. A blood smear was deemed negative only after examining 100 high-power microscopic fields. Hb levels were estimated using HemoCue.

### Randomizations and masking

The randomization codes were generated by an offsite statistician and randomization numbers were applied in sequence of recruitment. The trial was open-label. Participants and nurses performing home visits could not be masked. The study personnel at health facilities were necessarily aware of categories for assigning day of home visits, but were blind to the intervention arm.

### Anti-malarial treatment

In both arms children were treated with dispersible AL. The first dose was supervised at the health facility by a study nurse, and repeated if the child vomited within 30 min. The remaining five doses were taken at home. All caregivers received verbal instructions to give the second dose exactly 8 h after the first dose with the subsequent four doses given on the following 2 days at 08.00 in the morning and in the evening at 20.00, and illustrations on the AL blister packs that were taken home were used to support the explanations. Children weighing 5–14 kg were to take single tablet per dose while those weighing 15–24 kg two tablets per dose. The caregivers were advised to administer AL after a meal or with food, to complete all doses even if the child appeared better, and to return to the health facility immediately if the child’s condition worsened.

### Intervention—SMS reminders

In total 11 text messages were used where content, timing, understanding, and distribution had undergone extensive pre-testing with community members, caregivers and patients at four facilities within the same county but outside of the study area [37]. Caregivers in the intervention arm were sent automated SMS reminders, timed to start 8 h after the first AL dose and then every morning (08.00) and evening (20.00) until the full AL course was administered. For each post-treatment visit (i.e., on day 3 and day 28), two SMS reminders were sent, one in the evening prior to the day of the appointment and one in the morning on the day of the facility visit. Participants in category 3 were not sent the day-3 SMS reminder to come back to the facility because they would be visited on the same day at home. Three messages sent on days 7, 14 and 21 reminded caregivers about ‘unscheduled’ visits if child does not get better. The messages were sent in English, Kiswahili or Dholuo depending on caregivers’ language preferences. Table 1 shows the final content and delivery schedule of all text messages deployed.

**Table 1:** Content and schedule of SMS reminders

Message category	Timing	Day of sending	Message content
AL dose 2	8 h after first dose	Day 0	Hello [name of care giver], have you remembered to give your child the [dose number] dose of malaria medicine? If not, please do so. Thank you, [Name of HF]
AL dose 3	08:00	Day 1	
AL dose 4	20:00	Day 1	
AL dose 5	08:00	Day 2	
AL dose 6	20:00	Day 2	
Day 3 health facility post-treatment visit^a^	20.30	Day 2	Hello [name of care giver], please remember to bring the child back to hospital [tomorrow on day 2/today on day 3] to confirm clearance of malaria parasites. Thank you, [Name of HF]
	08:00	Day 3	
Unscheduled visit	08:00	Day 7	Hello [name of care giver] I hope the child is doing well. If not, please bring them back to the hospital as soon as possible. Thank you, [Name of HF]
	08:00	Day 14	
	08:00	Day 21	
Day 28 health facility post-treatment visit	18.30	Day 27	Hello [name of care giver], please bring your child back to the hospital [tomorrow on day 27/today on day 28] for day 28 post-treatment as advised by the doctor. Thank you, [Name of HF]
	08:00	Day 28	

^a^Day 3 post-treatment reminders are not sent to the patients in category 3 since they are visited at home

### Follow-up

During recruitment, caregivers were informed that they would be visited at home but not informed of the specific day of the visit. They were advised to keep AL blister packs after completing the treatment course. Home visits were undertaken by a study nurse within 24 h of expected completion of the individual doses of interest (doses 2 and 3 in category 1, doses 4 and 5 in category 2, and dose 6 in category 3). During the home visits, adherence to AL was assessed using pill counts and caregivers’ reports to determine the number of pills taken prior to the visit and the timing of each dose. Caregivers were also asked whether they had received text message reminders. Caregivers who returned to the health facility on day 3 (category 1 and 2) were not financially compensated for transport costs as this was considered routine, however those who returned to the facility on day 28 received travel compensation of approximately 2 USD.

### Outcomes and definitions

Two primary outcomes were investigated in the trial. The first outcome was the proportion of patients adhering to the complete AL course (doses 2–6) measured in category 3 using the combination of pill count and self-reporting. The definition of correct AL adherence was based on two criteria of which both had to be met: (1) completion of all doses; and, (2) correct timing of all doses. To assess completion of all doses the evidence of empty AL blister pack during the home visit was used. In the absence of blister packs, caregivers’ reports were used. To assess timing, caregivers’ report of administered doses, within ±1 h for dose 2 and ±2 h for doses 3–6, compared to the instructions given at the time of recruitment, was classified as correct. The second primary outcome was the proportion of patients in combined categories 1 and 2 who returned to the health facility on day 3 after expected completion of AL treatment.

Two secondary outcomes were also investigated in the trial. The first secondary outcome was the proportion of patients adhering to the individual AL doses measured within 24 h of expected dose administrations, i.e., for doses 2 and 3 in category 1, doses 4 and 5 in category 2, and for dose 6 in category 3. The adherence definitions for these patient groups followed the criteria of dose completion based on appropriate number of tablets found at the time of the visit and correct timing, using the same time allowances as described for the primary outcome. Finally, the last trial outcome was the proportion of patients across all categories that returned to the health facility on day 28.

### Sample size calculation

The sample size estimation was based on the first primary outcome (i.e., adherence to the full course of AL treatment in category 3) [38]. Assuming adherence of 65% in the control group, effect size of 10%, 15% loss to follow-up, 80% power and 0.05 level of significance, the estimated sample size was 400 participants per arm. In addition, 300 participants per arm were included in each of the first and second categories. These sample sizes were estimated to be sufficient to detect an effect size of 10% for an estimated individual AL dose adherence at 75% in the control group, assuming a 10% loss to follow-up and the same power assumptions. Finally, for the measurement of the day 3 return to health facilities, the combined sample size in categories 1 and 2 (600 per arm), had more than 90% power to detect a 10% difference from an estimate for the control group of 45% of patients returning on day 3. Due to slower recruitment than expected and on advice from the Trial Steering Committee, an interim analysis was conducted on the primary adherence outcome in category 3. The interim analysis found ~70% adherence in the control group, higher than the 65% initially estimated, and only 10% rather than 15% loss to follow-up. The sample size was therefore recalculated using other assumptions as above and the recruitment was terminated when 645 patients in category 3 and 1677 in total were enrolled.

### Analysis

The primary analytic approach was intention-to-treat (ITT) including all randomized patients with available outcome data. The secondary analytic approach was per-protocol (PP) where adherence analyses excluded patients who were inadvertently recruited without meeting enrolment criteria and those who did not receive SMS reminders in the intervention group. SMS exposure was classified on the basis of a caregiver’s report regarding delivery of any SMS reminder.

To estimate effects of the intervention on outcomes, mixed effects logistic regression models, with intervention arm as an independent variable adjusted for clustering by site, was used. The effect was expressed as an odds ratio (OR) with corresponding 95% confidence interval and *p* value. Participants’ characteristics were tabulated by randomization group. To further assess potential confounders, multivariable regression was performed by examining each potential confounder as an independent variable with randomization group and retaining any of the covariates, which changed the unadjusted OR of randomization group by more than 5%.

All trial data were double entered, verified and cleaned in Access 2013 (Microsoft Corporation, Seattle, WA), and thereafter analysed in Stata version 12 (StataCorp, College Station, TX).

## Results

### Enrolment and patient characteristics

Between 9 June, 2014 and 26 February, 2016, 8600 children presenting with suspected malaria were screened, of whom 1677 children were enrolled into the trial. The most common reasons for screen failures were 5387 (78.6%) malaria test-negative patients, 1335 (19.3%) without access to SMS messages, and 788 (11.4%) unwilling to comply with study protocol over 28 days (Fig. 1). Of the 1677 enrolled children, 849 (50.6%) were randomized into the control group and 829 (49.4%) into the intervention group. Analyses of the post-treatment return included all of the 1677 enrolled children, and for adherence measurements excluded 118 (7.0%) patients who were not found at home during the scheduled visits and 54 patients (3.7%) where visits were made but data on adherence were missing. The characteristics of the study population were similar between control and intervention groups among all enrolled patients overall and within categories (Table 2).

**Table 2:** The characteristics of the study subjects by arm and category

	Category 1		Category 2		Category 3		All patients	
	Control N = 254	Intervention N = 259	Control N = 262	Intervention N = 257	Control N = 333	Intervention N = 312	Control N = 849	Intervention N = 828
	n (%)	n (%)	n (%)	n (%)	n (%)	n (%)	n (%)	n (%)
Child characteristics								
Age (months)								
< 12	25 (9.8)	20 (7.7)	25 (9.5)	26 (10.1)	29 (8.7)	36 (11.5)	79 (9.3)	82 (9.9)
12–59	225 (88.6)	237 (91.5)	234 (89.3)	229 (89.1)	300 (90.1)	270 (86.6)	759 (89.4)	736 (88.9)
60	4 (1.6)	2 (0.8)	3 (1.2)	2 (0.8)	4 (1.2)	6 (1.9)	11 (1.3)	10 (1.2)
Male gender	143 (56.3)	144 (55.6)	136 (51.9)	129 (50.2)	175 (52.6)	163 (52.2)	454 (53.5)	436 (52.7)
Weight (kg)								
< 15	179 (70.5)	177 (68.3)	186 (71.0)	192 (74.7)	230 (69.1)	243 (77.9)	595 (70.1)	612 (73.9)
15–24	75 (29.5)	82 (31.7)	76 (29.0)	64 (24.9)	103 (30.9)	69 (22.1)	254 (29.9)	215 (26.0)
≥ 25	0	0	0	1 (0.4)	0	0	0	1 (0.1)
Temperature ≥37.5 °C	172 (67.7)	178 (68.7)	173 (66.0)	177 (68.9)	242 (72.7)	231 (74.0)	587 (69.1)	586 (70.8)
Parasite density >10,000/µl	188 (74.0)	197 (76.1)	196 (74.8)	187 (72.8)	263 (79.0)	224 (71.8)	647 (76.2)	608 (73.4)
Caregiver characteristics								
Age (years)								
≤20	41 (16.1)	62 (23.9)	54 (20.6)	57 (22.2)	56 (16.8)	63 (20.2)	151 (17.8)	182 (22.0)
20–40 years	189 (74.4)	182 (70.3)	195 (74.4)	181 (70.4)	251 (75.4)	238 (76.3)	635 (74.8)	601 (72.5)
>40	16 (6.3)	12 (4.6)	7 (2.7)	12 (4.7)	15 (4.5)	8 (2.6)	38 (4.5)	32 (3.9)
Missing age	8 (3.2)	3 (1.2)	6 (2.3)	7 (2.7)	11 (3.3)	3 (0.9)	25 (2.9)	13 (1.6)
Female gender	238 (93.7)	241 (93.1)	245 (93.5)	246 (95.7)	321 (96.4)	298 (95.5)	804 (94.7)	785 (94.8)
Relationship to the child								
Mother	225 (88.6)	224 (86.5)	242 (92.4)	233 (90.7)	292 (87.7)	292 (93.6)	759 (89.4)	749 (90.5)
Father	15 (5.9)	13 (5.0)	10 (3.8)	10 (3.9)	13 (3.9)	12 (3.9)	38 (4.5)	35 (4.2)
Other	14 (5.5)	22 (8.5)	10 (3.8)	14 (5.4)	28 (8.4)	8 (2.5)	52 (6.1)	44 (5.3)
Educational level								
No formal education	3 (1.2)	11 (4.3)	8 (3.0)	8 (3.1)	9 (2.7)	10 (3.2)	20 (2.4)	29 (3.5)
Primary	153 (60.2)	160 (61.8)	163 (62.2)	165 (64.2)	211 (63.4)	198 (63.5)	527 (62.0)	523 (63.2)
Secondary and above	97 (38.2)	87 (33.6)	88 (33.6)	84 (32.7)	111 (33.3)	104 (33.3)	296 (34.9)	275 (33.2)
Missing information	1 (0.4)	1 (0.3)	3 (1.2)	0	2 (0.6)	0	6 (0.7)	1 (0.1)
Phone ownership status								
Personal	193 (76.0)	209 (80.7)	188 (71.8)	193 (75.1)	259 (77.8)	237 (76.0)	640 (75.4)	639 (77.2)
Shared	61 (24.0)	50 (19.3)	74 (28.2)	64 (24.9)	74 (22.2)	75 (24.0)	209 (24.6)	189 (22.8)

### Adherence to complete AL course

Adherence to complete AL course was measured among 562 patients in category 3 (i.e., having home visits on day 3, Table 3); 92.4% of caregivers in the control group and 92.3% in the intervention group kept AL blister packs. The ITT analysis showed that 97.7% of patients completed all AL doses: 97.6% (282/289) in the control and 97.8% (267/273) in the intervention group (OR = 1.10; 95% CI = 0.37–3.33; p = 0.860). When defining adherence as completion of all doses and correct timing of all doses, 70.8% of the caregivers adhered to AL treatment schedule; 72.3% (209/289) in the control and 69.2% (189/273) in the intervention group (OR = 0.82; 95% CI = 0.56–1.19; p = 0.302). Adherence to individual AL doses in category 3 was high for all doses: 76.0% for dose 2; 96.3% for dose 3; 94.8% for dose 4; 95.6% for dose 5; and 89.9% for dose 6. No significant effect of the intervention on any of the five individual doses was observed (Table 3). PP analysis after excluding 62 patients who reported not receiving SMS in the intervention group and 14 protocol violations showed very similar results to the ITT analysis: 97.5% of patients completed all doses; 71.6% of the caregivers adhered to AL treatment schedule: 72.1% (204/283) in the control and 70.9% (144/203) in the intervention group (OR = 0.92; 95% CI = 0.61–1.38; p = 0.690), and without significant effects on any of the individual doses (Additional file 1).

**Table 3:** Effects of the intervention on AL adherence measured the day after expected completion of the full 3-day course—ITT analysis in category 3

AL adherence	Control N = 289 n (%)	Intervention N = 273 n (%)	All patients N = 562 n (%)	OR (95% CI)	p value
All AL doses completed	282 (97.6)	267 (97.8)	549 (97.7)	1.10 (0.37–3.33)	0.860
All doses timely completed	209 (72.3)	189 (69.2)	398 (70.8)	0.82 (0.56–1.19)	0.302
Dose 2					
Adherent	222 (76.8)	205 (75.1)	427 (76.0)	0.87 (0.58–1.29)	0.479
Dose 3					
Adherent	274 (94.8)	267 (97.8)	541 (96.3)	2.44 (0.93–6.37)	0.070
Dose 4					
Adherent	271 (93.8)	262 (96.0)	533 (94.8)	1.58 (0.73–3.41)	0.242
Dose 5					
Adherent	273 (94.5)	264 (96.7)	537 (95.6)	1.72 (0.75–3.96)	0.203
Dose 6					
Adherent	256 (88.6)	249 (91.2)	505 (89.9)	1.30 (0.74–2.28)	0.355

### Adherence to individual AL doses

Adherence to individual AL doses was measured in each of three categories of patients during home visits taking place within 24 h of expected completion of specific AL doses (Table 4). Very similar results were observed to those for the same measurements after completion of the 3-day course. No significant effect of the intervention was observed on adherence to any of the five AL doses examined (Table 4) and no significant effect of the intervention were observed on PP analysis (Additional file 2).

**Table 4:** Effects of the intervention on adherence to individual AL doses measured within 24 h of expected completion of the specific dose—ITT analysis by category

Doses	Patient category	Control n (%)	Intervention n (%)	All patients n (%)	OR (95% CI)	p value
Dose 2	1	N = 237	N = 241	n = 478		
Adherent		192 (81.0)	186 (77.2)	378 (79.1)	0.76 (0.48–1.19)	0.233
Dose 3	1					
Adherent		230 (97.1)	235 (97.5)	465 (97.3)	1.19 (0.39–3.59)	0.762
Dose 4	2	N = 226	N = 239	N = 465		
Adherent		205 (90.7)	223 (93.3)	428 (92.0)	1.43 (0.73–2.81)	0.303
Dose 5	2					
Adherent		207 (91.6)	220 (92.1)	427 (91.8)	1.06 (0.55–2.06)	0.857
Dose 6	3	N = 289	N = 273	N = 562		
Adherent		256 (88.6)	249 (91.2)	505 (89.9)	1.30 (0.74–2.28)	0.355

### Patients’ return to health facility

Patients’ return to health facility was assessed by combining data in categories 1 and 2 for day 3 return and then by combined data across all categories for day 28 return (Table 5). Of 1032 patients in categories 1 and 2, 81.4% of patients (420/516) in the intervention group and 74.0% of patients (382/516) in the control group returned to the facility on day 3. Similarly, among 1677 patients scheduled to return on day 28, 63.4% (525/828) returned in the intervention group and 52.5% (446/849) returned in the control group. Receiving the SMS intervention significantly increased odds of returning to the facility on day 3 (OR = 1.55; 95% CI = 1.15–2.08; p = 0:004) and on day 28 (OR = 1.58; 95% CI = 1.30–1.92; p < 0.001) (Table 5).

**Table 5:** Effects of the intervention on patients return to the health facility for post-treatment review

	Control n (%)	Intervention n (%)	All patients n (%)	OR (95% CI)	p value
Day 3 return	N = 516	N = 516	N = 1032		
(Patients from category 1 and 2)	382 (74.0)	420 (81.4)	802 (77.7)	1.55 (1.15–2.08)	0.004
Day 28 return	N = 849	N = 828	N = 1677		
(Patients from all categories)	446 (52.5)	525 (63.4)	971 (57.9)	1.58 (1.30–1.92)	<0.001

### Adjustment for covariates

No covariate met the 5% inclusion criteria described above for either outcome of interest (Additional files 3 and 4) and therefore only unadjusted effects of the intervention are presented, as above. Furthermore, no covariate showed statistically significant associations with outcome at p value of <0.10 on ITT or on PP analysis (Additional files 3 and 4).

## Discussion

This randomized controlled trial in western Kenya showed that SMS reminders significantly increased the rates of return to the health facility following anti-malarial treatment but did not have any effect on adherence to AL medications. The results of this efficacy trial comparing an SMS intervention to optimum clinical practices have several important implications.

Nearly all children completed all AL doses in this trial, which indicates substantially higher adherence than previously observed in evaluations conducted under the routine conditions in the same area [32, 33], in other parts of Kenya [39], and across Africa [40–45]. The high availability of AL blister packs during home visits and the evidence of empty packs provided confidence of high completion rates. Timely completion of all AL doses was lower (71%), but still substantially higher than reported in many previous studies [23, 40–42]. Adherence results for individual doses measured by home visits conducted closer to the expected time of administration were similar with measurements three days after the treatment. This suggests that self-reporting was unlikely to have introduced recall bias. While non-completion of AL doses compromises treatment outcome and contributes to the development of resistance [25, 46, 47] it is unclear how strictly dose intervals must be adhered to in order to avoid adverse consequences and this trial was not designed to examine this.

Two other previous effectiveness trials tested effects of innovative SMS reminders on patients’ adherence to anti-malarials and these showed discordant results, but lower overall adherence [22, 23]. Due to methodological differences where different modalities of SMS interventions were used in different settings, comparisons between studies are problematic. Under the trial conditions reported here, SMS reminders were unnecessary as adherence levels were already high. It is possible that the excluded patients from the trial were those in whom poor adherence was most likely. This can only be a partial explanation for high adherence rates, since relatively few caregivers were excluded simply because they declined to take part or lacked access to SMS reminders (most exclusions were for children without malaria).

A recent review of anti-malarial adherence studies suggests that interactions between research teams or medical staff and patients is likely to influence adherence levels [19]. Specifically, the conduct of consultations by research staff, parasitological confirmation, consenting of patients prior to the treatment, awareness of participants about home visits, dispenser’s observation of the swallowing of the first dose, may all have contributed to higher adherence levels in this trial. The importance of optimum clinical practices, including appropriate drug dispensing and counselling according to standard guidelines, either provided under the trial or routine conditions, seems to be a factor for adherence [48]. Under ‘real world’ conditions SMS reminders may lead to improved adherence to anti-malarial medicines [22]. However it can be argued that ‘real world’ conditions can be altered by the implementation of policy. For instance, healthcare providers in routine settings could provide explanations and demonstrate the illustrated instructions on blister packs as done in this trial. The investment required for SMS reminder systems should be compared against investment in the basic essentials and interventions to improve provider adherence to national treatment guidelines.

In contrast to the findings on adherence, SMS reminders increased the rates of patients returning to health facilities for routine follow-up. Increasing the day 3 return from 74 to 81% and the day 28 return from 53 to 63% is a relatively modest but still significant impact on behaviour. Two trials in Kenya have shown similar effects of SMS on postpartum [11] and postoperative [12] attendance, as well as a larger 18% effect on attendance for follow-up HIV testing [13]. Post-treatment attendances among patients not receiving SMS reminders were significantly higher than the range 12–41% observed in Kenyan appointment trials, as well as higher than the 44% of sick children returning to health facility following outpatient counselling in Sudan [21]. In the Kenyan trial reported in this manuscript, day 28 returns are likely to have been positively influenced by financial transport compensations and day 3 returns are likely to have been influenced by home visits. Nevertheless, the SMS reminder was seen to be significant in improving returns at both day 3 and day 28 despite this background. The costs and benefits of improved day 3 post-treatment returns among children receiving SMS reminders should be considered for routine use, particularly given the importance of pragmatic, artemisinin resistance monitoring [29, 30].

## Conclusions

When optimum care under the trial conditions is provided, text-message reminders can increase a child’s return to the health facility following anti-malarial treatment, without an additional effect on already high levels of AL adherence that occur under trial conditions. Further effectiveness studies under varying real world conditions in different settings are needed to determine the optimum role of text-message reminders in improving patients’ adherence to anti-malarial medicines.

## Additional files

**Additional file 1.** Effects of the intervention on AL adherence measured the day after expected completion of the full 3-day course—per-protocol analysis in category 3.

**Additional file 2.** Effects of the intervention on adherence to individual AL doses measured within 24 h of expected completion of the specific dose—per-protocol analysis by category.

**Additional file 3.** Association of factors with adherence to the full AL course and effects of potential confounders on the main study effect.

**Additional file 4.** Association of factors with return to facility and effects of potential confounders.
