# Supplementary material for: Efficacy of text-message reminders on paediatric malaria treatment adherence and their post-treatment return to health facilities in Kenya: a randomized controlled trial
Source: Malar J. 2017 Jan 25;16:46. doi: 10.1186/s12936-017-1702-6 (PMC5267364; doi:10.1186/s12936-017-1702-6)
Supplement: Supplementary file 1 — Additional file 1. Effects of the intervention on AL adherence measured the day after expected completion of the full 3-day course—per-protocol analysis in category 3. [file 12936_2017_1702_MOESM1_ESM.docx]

**Additional file 1 Effects of the intervention on AL adherence measured the day after expected completion of the full 3-day course – per-protocol analysis in category 3**

| **AL adherence** | **Control**  **N = 283** | **Intervention**  **N = 203** | **All patients**  **n = 486** | **OR (95% CI)** | **p-value** |
| --- | --- | --- | --- | --- | --- |
| **All AL doses completed** | 276 (97.5) | 198 (97.5) | 474 (97.5) | 1.00 (0.31-3.21) | 0.994 |
| **All doses timely completed** | 204 (72.1) | 144 (70.9) | 348 (71.6) | 0.92 (0.61-1.38) | 0.690 |
| **Dose 2** |  |  |  |  |  |
| Adherent | 217 (76.7) | 158 (77.8) | 375 (77.2) | 1.04 (0.67-1.62) | 0.855 |
| **Dose 3** |  |  |  |  |  |
| Adherent | 268 (94.7) | 198 (97.5) | 466 (95.9) | 2.22 (0.79-6.20) | 0.129 |
| **Dose 4** |  |  | ` |  |  |
| Adherent | 265 (93.6) | 195 (96.1) | 460 (94.7) | 1.66 (0.71-3.89) | 0.247 |
| **Dose 5** |  |  |  |  |  |
| Adherent | 267 (94.4) | 195 (96.1) | 462 (95.1) | 1.46 (0.61-3.48) | 0.393 |
| **Dose 6** |  |  |  |  |  |
| Adherent | 250 (88.3) | 184 (90.6) | 434 (89.3) | 1.26 (0.69-2.29) | 0.456 |
