# Supplementary material for: Efficacy of text-message reminders on paediatric malaria treatment adherence and their post-treatment return to health facilities in Kenya: a randomized controlled trial
Source: Malar J. 2017 Jan 25;16:46. doi: 10.1186/s12936-017-1702-6 (PMC5267364; doi:10.1186/s12936-017-1702-6)
Supplement: Supplementary file 2 — Additional file 2. Effects of the intervention on adherence to individual AL doses measured within 24 h of expected completion of the specific dose—per-protocol analysis by category. [file 12936_2017_1702_MOESM2_ESM.docx]

**Additional file 2 Effects of the intervention on adherence to individual AL doses measured within 24 hours of expected completion of the specific dose – per-protocol analysis by category**

| **Doses** | **Patient**  **Category** | **Control**  **n (%)** | **Intervention**  **n (%)** | **Total** | **OR (95% CI)** | **P-Value** |
| --- | --- | --- | --- | --- | --- | --- |
| **Dose 2** | **1** | **N = 225** | **N = 185** | **n = 410^a^** |  |  |
| Adherent |  | 184 (81.8) | 142 (76.8) | 326 (79.5) | 0.69 (0.42-1.12) | 0.136 |
| **Dose 3** | **1** |  |  |  |  |  |
| Adherent |  | 219 (97.3) | 181 (97.8) | 400 (97.6) | 1.20 (0.33-4.36) | 0.779 |
| **Dose 4** | **2** | **N = 219** | **N =198** | **N=417^b^** |  |  |
| Adherent |  | 198 (90.4) | 186 (93.9) | 384 (92.1) | 1.64 (0.79-3.44) | 0.186 |
| **Dose 5** | **2** |  |  |  |  |  |
| Adherent |  | 200 (91.3) | 185 (93.4) | 385 (92.3) | 1.35 (0.65-2.81) | 0.420 |
| **Dose 6** | **3** | **N = 283** | **N = 203** | **N =486^c^** |  |  |
| Adherent |  | 250 (88.3) | 184 (90.6) | 434 (89.3) | 1.26 (0.69-2.29) | 0.456 |

^a^ 68 patients excluded (18 protocol violations and 50 who reported not receiving SMS in the intervention group)

^b^ 48 patients excluded (9 protocol violations and 39 who reported not receiving SMS in the intervention group)

^c^ 76 patients excluded (14 protocol violations and 62 who reported not receiving SMS in the intervention group)
