# Supplementary material for: Efficacy of text-message reminders on paediatric malaria treatment adherence and their post-treatment return to health facilities in Kenya: a randomized controlled trial
Source: Malar J. 2017 Jan 25;16:46. doi: 10.1186/s12936-017-1702-6 (PMC5267364; doi:10.1186/s12936-017-1702-6)
Supplement: Supplementary file 3 — Additional file 3. Association of factors with adherence to the full AL course and effects of potential confounders on the main study effect. [file 12936_2017_1702_MOESM3_ESM.docx]

**Additional file 3 Association of factors with adherence to the full AL course and effects of potential confounders on the main study effect**

|  | **Bivariable intention to treat analysis** | | | | | | **Bivariable per-protocol analysis** | | | | | |
| --- | --- | --- | --- | --- | --- | --- | --- | --- | --- | --- | --- | --- |
|  | **N** | **n (%)**  **adherent** | **OR (95% CI)**  **of factor** | **p-value**  **of factor** | **aOR^a^ (95% CI)**  **intervention** | **p-value**  **of arm** | **N** | **n (%)**  **adherent** | **OR (95% CI)**  **of factor** | **p-value**  **of factor** | **aOR^a^ (95% CI)**  **of arm** | **p-value**  **of arm** |
| **Child characteristics** |  |  |  |  |  |  |  |  |  |  |  |  |
| **Age** |  |  |  |  |  |  |  |  |  |  |  |  |
| < 12 months | 59 | 39 (66.1) | Ref |  |  |  | 48 | 32 (66.7) | Ref |  |  |  |
| 12-60 months | 503 | 359 (71.4) | 1.42(0.78-2.57) | 0.247 | 0.83 (0.57-1.21) | 0.324 | 438 | 316 (72.2) | 1.43 (0.74-2.77) | 0.285 | 0.92 (0.61-1.39) | 0.701 |
| **Gender** |  |  |  |  |  |  |  |  |  |  |  |  |
| Female | 263 | 188 (71.5) | Ref |  |  |  | 231 | 166 (71.9) | Ref |  |  |  |
| Male | 299 | 210 (70.3) | 0.95 (0.65-1.39) | 0.798 | 0.82 (0.56-1.20) | 0.305 | 255 | 182 (71.4) | 0.98 (0.65-1.47) | 0.924 | 0.92 (0.61-1.38) | 0.691 |
| **Weight** |  |  |  |  |  |  |  |  |  |  |  |  |
| < 15kg | 411 | 296 (72.0) | Ref |  |  |  | 359 | 259 (72.1) | Ref |  |  |  |
| 15 – 25 kg | 151 | 102 (67.6) | 0.75 (0.50-1.15) | 0.186 | 0.80 (0.55-1.17) | 0.257 | 127 | 89 (70.1) | 0.85 (0.54-1.34) | 0.485 | 0.91 (0.60-1.37) | 0.648 |
| **Temperature** |  |  |  |  |  |  |  |  |  |  |  |  |
| ≥37.5 ⁰C | 410 | 288 (70.2) | Ref |  |  |  | 359 | 254 (70.8) | Ref |  |  |  |
| <37.5 ⁰C | 152 | 110 (72.4) | 1.14 (0.75-1.75) | 0.537 | 0.82 (0.56-1.20) | 0.305 | 127 | 94 (74.0) | 1.22 (0.76-1.94) | 0.411 | 0.93 (0.62-1.39) | 0.711 |
| **Parasite density** |  |  |  |  |  |  |  |  |  |  |  |  |
| >10,000/µl | 420 | 304 (72.4) | Ref |  |  |  | 365 | 269 (73.7) | Ref |  |  |  |
| ≤10,000/µl | 142 | 94 (66.2) | 0.85 (056-1.30) | 0.459 | 0.83 (0.57-1.21) | 0.335 | 121 | 79 (65.3) | 0.75 (0.48-1.18) | 0.215 | 0.94 (0.62-1.41) | 0.764 |
| **Caregiver characteristics** |  |  |  |  |  |  |  |  |  |  |  |  |
| **Age** |  |  |  |  |  |  |  |  |  |  |  |  |
| ≤ 20 years | 99 | 75 (75.8) | Ref |  |  |  | 82 | 63 (76.8) | Ref |  |  |  |
| 20 – 40 years | 4322 | 302 (69.9) | 0.77 (0.46-1.30) | 0.330 |  |  | 374 | 265 (70.9) | 0.76 (0.43-1.34) | 0.345 |  |  |
| > 40 years | 19 | 16 (84.2) | 2.19 (0.56-8.58) | 0.259 | 0.81 (0.55-1.18) | 0.273 | 18 | 15 (83.3) | 1.84 (0.46-7.31) | 0.385 | 0.91 (0.60-1.39) | 0.675 |
| **Gender** |  |  |  |  |  |  |  |  |  |  |  |  |
| Male | 22 | 15 (68.2) | Ref |  |  |  | 19 | 13 (68.4) | Ref |  |  |  |
| Female | 540 | 383 (70.9) | 1.11 (0.43-2.88) | 0.826 | 0.82 (0.56-1.20) | 0.306  0.306 | 467 | 335 (71.7) | 1.21 (0.44-3.34) | 0.717 | 0.92 (0.61-1.39) | 0.698 |
| **Relationship** |  |  |  |  |  |  |  |  |  |  |  |  |
| Mother | 510 | 363 (71.2) | Ref |  |  |  | 441 | 317 (71.9) | Ref |  |  |  |
| Other | 52 | 35 (67.3) | 0.84 (0.45-1.59) | 0.598 | 0.81 (0.56-1.18) | 0.281 | 45 | 31 (68.9) | 0.86 (0.43-1.71) | 0.668 | 0.91 (0.60-1.37) | 0.656 |
| **Educational level** |  |  |  |  |  |  |  |  |  |  |  |  |
| Primary and lower | 368 | 256 (69.6) | Ref |  |  |  | 320 | 228 (71.3) | Ref |  |  |  |
| Secondary and above | 192 | 140 (72.9) | 1.16 (0.78-1.73) | 0.459 | 0.83 (0.57-1.21) | 0.329 | 164 | 118 (72.0) | 1.02 (0.66-1.57) | 0.926 | 0.93 (0.62-1.40) | 0.725 |
| **Phone status** |  |  |  |  |  |  |  |  |  |  |  |  |
| Personal | 435 | 313 (72.0) | Ref |  |  |  | 390 | 281 (72.1) | Ref |  |  |  |
| Shared | 127 | 85 (66.9) | 0.91 (0.59-1.42) | 0.688 | 0.82 (0.56-1.20) | 0.304 | 96 | 67 (69.8) | 1.06 (0.63-1.76) | 0.833 | 0.92 (0.61-1.39) | 0.703 |

^a^ Adjusted Odds Ratio
