# Supplementary material for: Efficacy of text-message reminders on paediatric malaria treatment adherence and their post-treatment return to health facilities in Kenya: a randomized controlled trial
Source: Malar J. 2017 Jan 25;16:46. doi: 10.1186/s12936-017-1702-6 (PMC5267364; doi:10.1186/s12936-017-1702-6)
Supplement: Supplementary file 4 — Additional file 4. Association of factors with return to facility and effects of potential confounders. [file 12936_2017_1702_MOESM4_ESM.docx]

**Additional file 4 Association of factors with return to facility and effects of potential confounders**

| **Bivariable ITT analysis** | **N** | **n (%)**  **return to facility** | **OR (95% CI)**  **of factor** | **p-value**  **of factor** | **aOR^a^ (95% CI)**  **intervention** | **p-value**  **of arm** |
| --- | --- | --- | --- | --- | --- | --- |
| **Child characteristics** |  |  |  |  |  |  |
| **Age** |  |  |  |  |  |  |
| < 12 months | 96 | 73 (76.0) | Ref |  |  |  |
| 12-60 months | 936 | 729 (77.9) | 1.16 (0.70-1.90) | 0.571 | 1.55 [1.15-2.08] | 0.004 |
| **Gender** |  |  |  |  |  |  |
| Female | 480 | 382 (79.6) | Ref |  |  |  |
| Male | 552 | 420 (76.1) | 0.82 (0.61-1.10) | 0.187 | 1.55 [1.15-2.08] | 0.004 |
| **Weight** |  |  |  |  |  |  |
| < 15kg | 734 | 566 (77.1) | Ref |  |  |  |
| 15 – 25 kg | 298 | 236 (79.2) | 1.17(0.84-1.62) | 0.368 | 1.55 [1.15-2.09] | 0.004 |
| **Temperature** |  |  |  |  |  |  |
| ≥37.5 ⁰C | 700 | 541 (77.3) | Ref |  |  |  |
| <37.5 ⁰C | 332 | 261 (78.6) | 1.07 (0.78-1.48) | 0.670 | 1.55 [1.15-2.09] | 0.004 |
| **Parasite density** |  |  |  |  |  |  |
| >10,000/µl | 768 | 589 (76.7) | Ref |  |  |  |
| ≤10,000/µl | 264 | 213 (80.7) | 1.25 (0.88-1.77) | 0.218 | 1.55 [1.15-2.09] | 0.004 |
| **Caregiver characteristics** |  |  |  |  |  |  |
| **Age** |  |  |  |  |  |  |
| ≤ 20 years | 214 | 164 (76.6) | Ref |  |  |  |
| 20 – 40 years | 747 | 582 (77.9) | 1.14 (0.79-1.64) | 0.488 |  |  |
| > 40 years | 47 | 37 (78.7) | 1.24 (0.57-2.70) | 0.591 | 1.57 [1.16-2.12] | 0.003 |
| **Gender** |  |  |  |  |  |  |
| Male | 62 | 47 (75.8) | Ref |  |  |  |
| Female | 970 | 755 (77.8) | 1.10 (0.60-2.01) | 0.766 | 1.55 [1.15-2.08] | 0.004 |
| **Relationship** |  |  |  |  |  |  |
| Mother | 924 | 722 (78.1) | Ref |  |  |  |
| Other | 108 | 80 (74.1) | 0.81 (0.51-1.28) | 0.359 | 1.55 [1.15-2.09] | 0.004 |
| **Educational level** |  |  |  |  |  |  |
| Primary and lower | 671 | 524 (78.1) | Ref |  |  |  |
| Secondary and above | 356 | 276 (77.5) | 0.95 (0.69-1.30) | 0.734 | 1.55 [1.15-2.09] | 0.004 |
| **Phone status** |  |  |  |  |  |  |
| Personal | 783 | 613 (78.3) | Ref |  |  |  |
| Shared | 249 | 189 (75.9) | 0.90 (0.64-1.26) | 0.530 | 1.54 [1.14-2.08] | 0.005 |

^a^ Adjusted Odds Ratio
